# Elevated levels of exhaled nitric oxide in patients with anorexia nervosa

**DOI:** 10.1007/s00787-013-0467-x

**Published:** 2013-11-26

**Authors:** Joanna Oświęcimska, Katarzyna Ziora, Dariusz Ziora, Edyta Machura, Sebastian Smerdziński, Magdalena Pyś-Spychała, Jacek Kasperski, Jacek Zamłyński, Alicja Kasperska-Zajac

**Affiliations:** 1 Department of Paediatrics, Medical University of Silesia in Katowice, ul. 3 Maja 13/15, 41-800 Zabrze, Poland; 2 Department of Pneumonology and Tuberculosis, Medical University of Silesia in Katowice, ul. Koziołka 1, 41-803 Zabrze, Poland; 3 Department of Paediatrics, Regional Hospital in Strzelce Opolskie, ul. Opolska 36A, 47-100 Strzelce Opolskie, Poland; 4 Department of Prosthetic Dentistry, Medical University of Silesia in Katowice, ul Plac Akademicki 17, 41-902 Bytom, Poland; 5 Department of Gynaecology, Obstetrics and Oncological Gynaecology, Medical University of Silesia in Katowice, ul Batorego 15, 41-902 Bytom, Poland; 6 Department of Internal Diseases, Dermatology and Allergology, Medical University of Silesia in Katowice, ul. Marii Curie-Skłodowskiej 10, 41-800, Zabrze, Poland

**Keywords:** Nitric oxide, Anorexia nervosa, Exhaled NO, Niox Mino^®^ analyzer, Acute phase response

## Abstract

**Background:**

Nitric oxide (NO) is involved in eating behavior and inflammatory response. Moreover, there is evidence that NO production is altered in patients with anorexia nervosa (AN).

**Aim:**

To assess whether the overproduction of NO in AN can affect NO level in exhaled air.

**Materials and methods:**

Exhaled NO level was studied in 23 girls with AN and compared with that of healthy age- and gender-matched nonatopic controls.

**Results:**

Exhaled NO levels were significantly higher in girls with AN compared with healthy age-matched controls.

**Conclusions:**

It appears that anorexia nervosa was accompanied by a higher level of exhaled NO, likely resulting from a systemic increase in NO production because of the severe catabolic state.

## Introduction

Anorexia nervosa (AN) is a complex syndrome characterized by changes in eating behavior, and its etiopathogenesis remains poorly understood. Various mediators have been considered to play an important role in appetite regulation and food intake, including nitric oxide (NO) [1].

It is known that NO plays a role in body energy balance by modulation of mitochondrial biogenesis [2] and proliferation/differentiation of adipocytes [3]. In addition, upregulation of NO synthesis by inducible nitric oxide synthase (iNOS) may induce injury and self-destructive processes.

Interestingly, increased NO production and expression of iNOS have been demonstrated in patients with AN, suggesting enhanced NO production in the disease [1].

Exhaled NO is produced in a large amount in patients with inflammatory lung diseases, including asthma and can be a sensitive, yet nonspecific marker of disease activity [4–6]. The level of exhaled NO may be increased in many other diseases, including bronchiectasis [7] chronic tonsillitis [8], infections of upper respiratory tract [9], and lupus [10]; however, it was not shown to be a reliable biomarker in glottic carcinoma [11].

To date, it has not been determined whether the overproduction of NO in AN can affect NO level in exhaled air. In contrast to serum NO and iNOS expression, exhaled NO can be easily measured using commercially available equipment thereby facilitating further studies of its role in the pathogenesis of eating disorders. Thus, in the current study we measured exhaled oral NO in girls with AN and in healthy age- and gender-matched nonatopic controls.

## Materials and methods

The study involved 23 girls (mean age 16.0 ± 1.2 years, range 12.1–17.7) who, following pediatric examination, psychological evaluation, and psychiatric consultation, were diagnosed with AN in accordance with the classification in the American Psychiatric Association’s *Diagnostic and Statistical Manual of Mental Disorders* *IV*. The patients were assessed using semi-structured interview (i.e., The Eating Disorder Examination) [12]. Expected body weight was established using normal ranges for the Polish population of girls [13].

Mean duration of the disease before hospitalization was 16.5 ± 17.7 months. The evaluation was based on analysis of hospital medical documentation regarding patients with AN referred to the Department of Pediatrics and Endocrinology before re-feeding treatment. The patients did have concomitant diseases (screened by clinical history, physical examination, electrocardiography, chest radiography, spirometry, and urine and blood tests), either severe somatic complications of AN (gastrointestinal bleeding, dehydration, peptic ulcer disease, liver or kidney dysfunction), or psychiatric disorders. All subjects were Tanner stage IV–V. On recruitment, none of the patients were taking drugs (including anti-anxiety medications or psychotropic drugs). During hospitalization patients were placed on bed rest, were fed a high-calorie diet, and received psychotherapy.

Routine allergological, dental and laryngological examinations were performed. Exclusion criteria consisted of the known diseases and other factors that may affect exhaled NO concentration, including asthma, atopy, rhinosinusitis and dental caries. In addition, patients with recurrent respiratory symptoms or respiratory infection in the preceding 3 months and smokers were excluded from the study.

Spirometric parameters are decreased in patients with AN, as explained by diminished respiratory muscle force, mainly in the diaphragm, due to weight loss [14]. Therefore, the Niox Mino^®^ analyzer test was routinely performed during the first 2 days after admission of such patients in our center [14].

The control group consisted of 13 age-matched (mean age 15.2 ± 1.3 years; range 13.1–17.4), non-smoking girls without signs of atopy and asthma, and with normal spirometry results.

The subjects were instructed to avoid food that contained high levels of nitrites or nitrates. The tests were performed between 8 and 9 a.m. in a fasting state.

Exhaled NO concentrations were correlated with anthropometric data: body weight, height, body mass index (BMI), spirometric parameters (forced expiratory volume in 1 s [FEV1], Tiffeneau–Pinelli index [FEV1/FVC]), erythrocyte sedimentation rate (ESR), serum C-reactive protein (CRP), and total immunoglobulin E (IgE) concentrations.

The study was conducted according to the Declaration of Helsinki and approved by the Bioethics Committee of the Medical University of Silesia in Katowice (Registry No KNW/0022/KB/211/12). Informed written consent for participation in the study was obtained from all study subjects and their parents or legal guardians.

The clinical characteristics of examined groups are presented in Table 1.

**Table 1:** Clinical characteristics of the examined groups

Parameter	AN (*n* = 23)	Controls (*n* = 16)	*P* value
Age (years)	16.0 ± 1.2	15.2 ± 1.3	0.06
Body weight (kg)	42.7 ± 4.9	54.6 ± 9.2	<0.001*
Height (cm)	162.9 ± 4.3	160.9 ± 6.2	0.24
BMI (kg/m^2^)	16.0 ± 1.6	21.1 ± 3.1	<0.001*
ESR (mm/h)	6.6 (5.0–8.0)	6.4 (3.0–10.0)	0.71
C-reactive protein (mg/l)	0.4 (0.3–0.7)	0.5 (0.3–0.7)	0.86
Total IgE (IU/ml)	21.6 (10.9–68.2)	30.4 (17.2–31.7)	0.79
FEV1 (%)	92.7 ± 21.0	96.6 ± 12.0	0.56
FEV1/FVC (%)	88.6 ± 10.8	94.3 ± 8.8	0.12

Data expressed as mean ± SD for normally distributed variables (age, body mass, height, BMI, FEV1, FEV1/FVC) and as medians and quartiles for not normally distributed ones (OB, CRP, total IgE)

*BMI* body mass index, *ESR* erythrocyte sedimentation rate, *FEV1* forced expiratory volume in 1 s, *FVC* forced vital capacity

* *P* < 0.05

### Measurement of NO

Exhaled oral NO levels were measured in triplicate using Niox Mino^®^ analyzer (Aerocrine AB, Solna, Sweden). The level of NO is expressed in parts per billion (ppb).

### Statistical Analysis

The database was prepared using Excel 2000 (Microsoft Corporation). Statistical analysis was carried out with Statistica 6.0 software (StatSoft Inc., Tulsa, Oklahoma, USA). Normal data distribution was assessed using the Shapiro–Wilk test; the homogeneity of variance was computed using Levene’s test. Results are presented as mean ± standard deviation (SD) for normally distributed variables: age, body mass, height, BMI, NO, FEV1, FEV1/FVC and as medians and quartiles for non-normal distribution: ESR, CRP, and total IgE. The Student’s *t* test or the Mann–Whitney *U* test (if distribution of data was different from normal) was used for intergroup comparisons. Spearman’s correlation coefficients were used to estimate linear relationships between variables. *P* values <0.05 were considered significant.

## Results

**Fig. 1:**
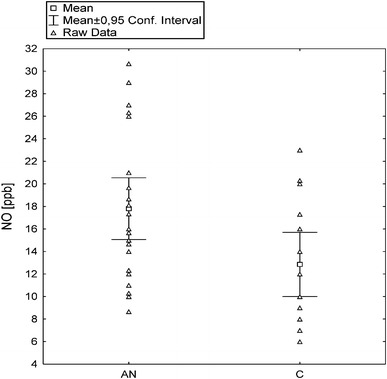
Exhaled NO levels in the examined groups: *AN* anorexia nervosa, *C* healthy controls (*P* < 0.05)

Exhaled NO levels were significantly higher (*P* < 0.05) in girls with AN (mean: 17.8 ± 6.3 ppb, range 8.6–30.6) than in healthy controls (mean: 12.8 ± 5.3 ppb; range: 6.0–23.0), (Fig. 1). Anorectic subjects had significantly lower (*P* < 0.001) body weight and BMI compared with the control group (42.7 ± 4.9 vs. 54.6 ± 9.2 kg and 16.0 ± 1.6 vs. 21.1 ± 3.1 kg/m^2^, respectively), but there were no differences in other clinical parameters (Table 1). There were no significant correlations between exhaled NO levels and serum CRP concentration, BMI, FEV1, FEV1/FVC or disease duration in the AN group. However, we observed a weak, but significant positive correlation with ESR value (*r* = 0.38; *P* < 0.05) in these patients (Table 2).

**Table 2:** Spearman`s linear correlations between exhaled NO values [ppb] and other clinical parameters in the examined groups

	*P* value		
	AN (*n* = 23)	Controls (*n* = 16)	All (*n* = 39)
Age (years)	0.01	0.26	0.20
Body weight (kg)	−0.11	0.28	−0.17
Height (cm)	0.19	0.56*	0.44*
BMI (kg/m^2^)	−0.29	0.21	−0.33*
Duration of the disease (months)	0.14	–	–
ESR (mm/h)	0.38*	0.89*	0.51*
C-reactive protein (mg/l)	0.01	0.07	0.01
Total IgE (IU/ml)	−0.29	0.11	−0.09
FEV1 (%)	0.14	0.50*	0.12
FEV1/FVC (%)	−0.14	0.64*	−0.01

*BMI* body mass index, *ESR* erythrocyte sedimentation rate, *FEV1* forced expiratory volume in 1 s, *FVC* forced vital capacity

* *P* < 0.05

Conversely, in healthy controls exhaled NO levels correlated positively with height (*r* = 0.56; *P* < 0.05), ESR (*r* = 0.89; *P* < 0.05), FEV1 (*r* = 0.50; *P* < 0.05), and FEV1/FVC (*r* = 0.64; *P* < 0.05) (Table 2).

Considering all examined subjects, we found a positive and significant relationship between exhaled NO levels and height (*r* = 0.44; *P* < 0.05) and ESR (*r* = 0.51; *P* < 0.05). Moreover, a negative correlation between exhaled NO and BMI was found (*r* = −0.33; *P* < 0.05) (Table 2).

## Discussion

This is the first evidence of increased exhaled NO level in patients with AN. These results extend and confirm previous observations that pointed to enhanced NO production––such as levels of NO metabolites in plasma [1] and in platelets [15]—among patients with AN. The NO overproduction found in AN platelets might be due to iNOS upregulation [15]. However, reports in the literature are scarce and contain conflicting data on the pattern of NO behavior in AN. In contrast to the aforementioned studies [1, 15], Rodrigues-Pereira et al. [16] demonstrated diminished NO production in patients with AN, as evaluated by NOS activity and cyclic guanosine monophosphate (cGMP) in platelets.

There is also some evidence suggesting that NO modulates the function of hypothalamic arcuate nucleus (ARC) in an animal model of sickness anorexia induced by bacterial endotoxin lipopolysaccharide (LPS) injection [17]. iNOS-dependent NO formation inhibits orexigenic NPY-containing arcuate neurons that are excited by ghrelin. This mechanism may be responsible for chronic anorexia in the course of inflammatory diseases, such as bacterial or parasitic infections, cancer, AIDS, kidney and heart failure, rheumatoid arthritis, and chronic obstructive pulmonary disease [18]. It also explains the weak efficiency of externally administrated potent orexigenic factor ghrelin in cancer anorexia–cachexia. Interestingly, ghrelin levels in AN are markedly increased, supporting the concept that in chronic food restriction (and probably in AN), a dysregulation of the agouti-related protein (AgRP) system contributes to deficient ghrelin signaling at the level of the ARC [19]. One can speculate that as a potent neuromodulator, NO may be involved in this phenomenon.

Apart from systemic and platelet levels, NO can be easily measured in exhaled air as an inflammatory marker in the respiratory tract, where its concentration depends on local production by vascular endothelium, smooth muscles, epithelium, macrophages, neutrophils, and fibroblasts. It is well known that in asthmatic patients increased exhaled NO may reflect enhanced iNOS expression in different airway cells resulting from the action of proinflammatory cytokines [5]. In contrast, there is lack of information regarding NOS/NO/cGMP system function in the lungs of patients with AN.

In our patients, bronchial asthma and other diseases of the respiratory tract associated with increased level of exhaled NO were excluded. However, even in the absence of respiratory diseases some spirometric abnormalities of lung function tests have been reported in patients with AN, including diminished FEV1, likely resulting from respiratory muscle weakness and body mass loss [14]. In the present study, we did not observe significantly decreased FEV1 in patients with AN compared with healthy subjects. In addition, there was no significant association between values of FEV1 and NO in patients with AN.

Interestingly, it has been demonstrated that NO production is increased in systemic diseases, including lupus, without concomitant symptoms from the airways [10] indicating that the diseases associated with increased systemic upregulation of NO pathway may also influence exhaled NO level.

It has been also suggested that the level of NO in the exhaled air may increase in response to inflammatory mediators released in sites other than the respiratory tract [10, 20].

AN may be associated with different neuroendocrine and immuno-inflammatory changes affected by the catabolic state. The role of impairment of the pro-inflammatory cytokine network in the development of AN has been proposed [21, 22], but data on circulating levels of proinflammatory cytokines in AN are conflicting.

In our study no association was found between serum CRP concentration and exhaled NO level. CRP was used as a marker reflecting the systemic effects of pro-inflammatory cytokines associated with acute phase response [23].

We observed a weak, but significant positive correlation between ESR value and exhaled NO level both in anorectic and control groups. The reason for this association remains unknown and cannot be clearly explained due to scarce data in the literature. There are several hypothetical explanations for this result. Both ESR and CRP values in our subjects were within normal range. Also, they did not show any clinical signs of infection. It is known that ESR is a sensitive yet nonspecific marker of an inflammatory state and cancer. It depends on albumin, γ- and α-globulin as well as fibrinogen concentrations. In addition, it may be influenced by red blood count and erythrocyte size, physiochemical parameters of blood, and other factors with unknown roles. Interestingly, Berg et al. [24] demonstrated that in isolated rabbit lungs perfused with hemoglobin or erythrocytes, concentrations of NO in the expired gas are decreased in comparison with lungs perfused with buffer as a result of hemoglobin NO scavenging. They also showed the influence of pulmonary microcirculation on exhaled NO levels. Therefore, we can speculate that the correlation between levels of exhaled NO and ESR may result rather from physiochemical and rheological properties of blood than the presence of the inflammatory state.

In contrast, another study did not find a correlation between exhaled NO and elevated ESR in patients with pulmonary tuberculosis [25]. Furthermore, this association was not investigated in healthy subjects [25]. To the best of our knowledge, data on the correlation in a large sample of healthy subjects and patients with different diseases do not exist. Finally, our study had a very small sample size; if a larger sample size was investigated, it would have resulted in a better representation of the population and quite possibly a more concise explanation of the possible mechanisms. Further studies are needed to clarify this problem.

In our study, exhaled NO levels correlated with the height of examined control subjects. Our findings are in contrast with previously published observations in children [26, 27]. However, in the study by van der Haijeden et al. [26] the examined children were much younger than our subjects and they used the offline tidal breathing method.

On the other hand, the systematic review of research on the fraction of exhaled NO reference values and the individual-specific factors that influence them demonstrated that height was significant factor in 7 of 15 eligible studies [28]. The airway diffusing capacity for NO is theoretically dependent on the airway mucosal surface area that has been shown to correlate with anatomic dead space volume in healthy children [29]. Thus, it is logical that age and height were found to be important factors when evaluating exhaled NO values, especially in this group of subjects. Moreover, similar results in teenagers were demonstrated by Linn et al. [30]. The lack of the above-mentioned correlation in AN may reflect the disturbed biological control of NO production in the airways due to negative energetic balance in the disease.

Taken together, AN may be characterized by enhanced production of NO resulting in an increase in circulating NO level, which is accompanied by parallel changes in exhaled NO induced by self-destructive catabolic processes. Interestingly, we found a weak negative correlation between exhaled NO and BMI in the examined subjects. Further studies are needed to assesses whether an impairment of NO pathway plays a role in the pathogenesis of AN.

### Limitations

Firstly, the relatively small and unbalanced sample size could be a potential drawback. Secondly, the repeated measurement of exhaled NO in the same patients, before therapy and after weight gain, seems essential for exact interpretation of our results. Thirdly, we did not determine the source of enhanced NO release in the airways of patient with AN. It might depend on local production within the respiratory tract. Therefore, further investigation of lung NO pathway in AN, such as iNOS expression, is needed.

### Clinical relevance

Our study provides evidence that in eating disorders, such as AN, the level of exhaled NO is higher than that of healthy persons. This finding should be considered, especially during diagnostic and therapeutic decisions regarding concurrent lung diseases, including bronchial asthma. In addition, taking into account previous data, a possible role of NOS/NO/cGMP system in the pathogenesis of eating disorders is suggested [17, 18].

## Conclusions

In conclusion, NO levels in exhaled air are significantly increased in girls with AN compared with the healthy age- and gender-matched controls. It seems that AN is accompanied by a higher level of exhaled NO, likely resulting from a systemic increase in NO production because of the severe catabolic state, although the clinical significance of this observation is unclear. Whether the increased NO production contributes to the pathogenesis of AN or is merely a secondary consequence, calls for future investigation. Further studies are also needed to clarify sources of the increased NO level in exhaled air.
